# Deep‐neural network approaches for predicting 3D dose distribution in intensity‐modulated radiotherapy of the brain tumors

**DOI:** 10.1002/acm2.14197

**Published:** 2023-11-07

**Authors:** Maziar Irannejad, Iraj Abedi, Vida Darbaghi Lonbani, Maryam Hassanvand

**Affiliations:** ^1^ Department of Electrical Engineering, Najafabad Branch Islamic Azad University Najafabad Iran; ^2^ Medical Physics Department, School of Medicine Isfahan University of Medical Sciences Isfahan Iran; ^3^ Department of Physics Isfahan University of Technology Isfahan Iran

**Keywords:** brain tumors, deep learning, dose prediction, IMRT

## Abstract

**Purpose:**

The aim of this study is to reduce treatment planning time by predicting the intensity‐modulated radiotherapy 3D dose distribution using deep learning for brain cancer patients. “For this purpose, two different approaches in dose prediction, i.e., first only planning target volume (PTV) and second PTV with organs at risk (OARs) as input of the U‐net model, are employed and their results are compared.”

**Methods and Materials:**

The data of 99 patients with glioma tumors referred for IMRT treatment were used so that the images of 90 patients were regarded as training datasets and the others were for the test. All patients were manually planned and treated with sixth‐field IMRT; the photon energy was 6MV. The treatment plans were done with the Collapsed Cone Convolution algorithm to deliver 60 Gy in 30 fractions.

**Results:**

The obtained accuracy and similarity for the proposed methods in dose prediction when compared to the clinical dose distributions on test patients according to MSE, dice metric and SSIM for the Only‐PTV and PTV‐OARs methods are on average (0.05, 0.851, 0.83) and (0.056, 0.842, 0.82) respectively. Also, dose prediction is done in an extremely short time.

**Conclusion:**

The same results of the two proposed methods prove that the presence of OARs in addition to PTV does not provide new knowledge to the network and only by defining the PTV and its location in the imaging slices, does the dose distribution become predictable. Therefore, the Only‐PTV method by eliminating the process of introducing OARs can reduce the overall designing time of treatment by IMRT in patients with glioma tumors.

## 1 INTRODUCTION

In recent years, due to the increasingly advances in the quality of radiotherapy methods, techniques such as intensity‐modulated radiation therapy (IMRT (and volumetric‐modulated arc therapy (VMAT) have become standard treatments for brain tumors.^1^, ^2^, ^3^, ^4^, ^5^, ^6^ These high‐quality radiotherapy methods aim to deliver a high‐radiation dose to the tumor while preserving the organs at risk (OARs).^7^, ^8^ In IMRT, the plan optimization is done by a computer method called Inverse Planning which is based on objective functions and dose constraints.^9^, ^10^ For this purpose, the planner uses the trial‐and‐error method for parameter optimization to meet the optimal treatment plan. This optimization is very time‐consuming and depends on the planner's experience and skills.^11^, ^12^

Various research has been conducted to automate treatment planning to improve the quality of treatment and reduce planning time.^13^ Dose‐volume histogram (DVH) is a common criterion of inverse planning, however, this method does not contribute to spatial information.^14^, ^15^ Atlas‐based methods use the selection of the nearest matching patient to find a better starting point for inverse optimization.^16^ In some methods, the optimization is based on voxel dose constraints.^17^, ^18^ Although these methods apply automated optimization and are based on constraints, they still need different tunings and several time executions to achieve their desired goals. Recently, deep learning algorithms in artificial intelligence have improved visual data analysis, allowing for the results to be compared with state‐of‐the‐art modern methods.^19^, ^20^, ^21^

A convolutional neural network (CNN) within deep learning makes it possible to extract different levels of features (low, mid, high) automatically and without any manual intervention.^22^

In recent years, efforts have been made to use deep learning to predict the dose distribution in tomotherapy for prostate,^23^, ^24^, ^25^, ^26^ left‐side breast,^27^ lung,^28^ and head and neck cancers^29^, ^30^, ^31^, ^32^, ^33^, ^34^ to reduce overall planning time and manual planning.

In this study, we present a method based on artificial intelligence to predict the dose distribution for brain tumors treated by IMRT. In other words, to increase the speed and accuracy of treatment planning through deep learning, we seek to extract the existing knowledge of treated patients and applied it to treat new patients.

As the novelty of this work, according to our studies, no research has been done so far in predicting the dose of brain tumors treated by IMRT using deep learning. Additionally, two different approaches were tested for dose prediction: only PTV and PTV‐OARs methods, and found that the former significantly enhances the planning speed compared to the latter.

## 2 MATERIALS AND METHODS

### 2.1 Patient data

The total of 99 patients with glioma tumors referred for IMRT treatment between August 2018 and November 2020 were included in this study. All patients had T13N0M0 clinical‐stage glioma tumors and were classified as high‐risk as defined by the National Comprehensive Cancer Network (www.nccn.com). Patients were subjected to a CT simulation with immobilization using a custom thermoplastic mask system. For better delineation of the target and OARs, the patient's MRI was fused with the non‐contrast CT scan using the PROWESS Panter (Version 5.5) treatment planning system (TPS). Gross target volume (GTV) was defined as the resection cavity plus any contrast‐enhancing area on a post‐gadolinium T1‐weighed MRI. Clinical target volume (CTV) was obtained by adding a three‐dimensional 1 cm expansion to the GTV. The CTV was then expanded by 1 cm to create the PTV. Contoured OARs were the brainstem, optic nerve, optic chiasm, lenses, eye globes, and healthy brain. The patients were all manually planned and treated with sixth‐field IMRT; the photon energy was 6 MV, and the gantry angles were 60°, 100°, 165°, 195°, 260°, and 300°. All of the treatment plans were done with the Collapsed Cone Convolution (CCC) algorithm to deliver 60 Gy over 30 fractions. The plans were acceptable when 95% of the prescription dose covered 99% or more of the PTV. All the critical structures including optic chiasm, optic nerves, eye globes, brainstem, and lenses can be considered as serial structures, hence, planning constraints were referred to as the maximum dose. The dosimetric constraints and priorities leading the planning optimization for OARs were defined as follows:

Brainstem (D_max_ ≤ 54 Gy), Cochlea (D_mean_ ≤ 45 Gy), Cortex (D_max_ ≤ 28.6 Gy), Eyes (D_max_ ≤ 45 Gy), Lens (D_max_ ≤ 6 Gy) and Optic nerves (D_max_ ≤ 54 Gy).

For each plan, the contours of the PTV and the OARs were determined by experienced radiation oncologists while the dose distribution was optimized and confirmed by experienced medical physicists.

### 2.2 Model architecture

In this study, a U‐net network has been implemented to achieve three‐dimensional contours for dose distribution prediction. The U‐Net is a convolutional neural network originally proposed for medical imaging segmentation.^20^, ^21^, ^22^ Recently U‐net and its derivatives have been used as a model to predict dose distribution in tomotherapy.^23^, ^24^, ^25^, ^26^, ^27^, ^28^, ^29^, ^30^, ^31^, ^32^, ^33^, ^34^

The U‐net network configuration consists of three main components: the encoder, decoder, and skip‐connections. In the encoder part, which is a contraction path where the image content is extracted. In this path, the spatial scale of the image decreases and the number of feature mapping layers increases. In the decoder, which is an expansion path symmetrical to the encoder, the output resolution is enhanced step by step. In order to locate features that have been scaled up, skip‐connections are utilized to transfer the features extracted through the encoder to the decoder. Each of the encoder and decoder paths consists of four stages. Each stage consists of two 3 × 3 × 3 convolutional layers followed by a batch normalization (BN) layer and the rectified linear unit (ReLU). Passing each stage was done with 2 × 2 × 2 max‐pooling layers for the down‐sampling process in the encoder and 2 × 2 × 2 deconvolution layers for the up‐sampling process in the decoder. To train the model, binary cross entropy (BSC) is used as a loss function that compares the similarity between the voxel of prediction with the clinical dose. Also, a dice metric with a 1 × 10^−5^ smooth factor was combined with BSC to test the performance of prediction. The dice metric by calculating the amount of overlap between the predicted dose and clinical dose helps to obtain better model performance. The adaptive moment estimation (Adam) algorithm by 3 × 10^−4^ learning rate with a batch size of 2 is employed to optimize the weights or parameters during the training process.

In this study, in order to predict the dose distribution in radiotherapy of brain tumors, two approaches were applied and compared. In the first method, only the images containing the PTV (without OARs) were considered to predict the dose, and in the second one, in addition to the PTV, the images of four main organs (PTV‐OARs), including the eyes, lens, optic nerves, and optic chiasm were taken into account. It is worth noting here that in both methods the skin was also regarded as a marker to determine the location of PTV and OARs in the different slices of the brain image. In the first method, the network is trained using two‐channel (white and black) images as the input. But in the second method, in order to separate the OARs and PTV from each other, each element is identified with a specific color, or in other words, it is introduced to the network in three‐channel images (RGB mode). Since the percentages of different doses were trained separately in the output of the network, two‐channel images corresponding to the input image slices were regarded as the targets. In this study, the images from 99 patients treated by IMRT were considered, so that the images from nine patients were employed for the training and validation set in 5‐fold cross‐validation, and the residual I mages from 9 patients were used to evaluate the network performance. The classification of patient images for training and the test set was done randomly.

**FIGURE 1:**
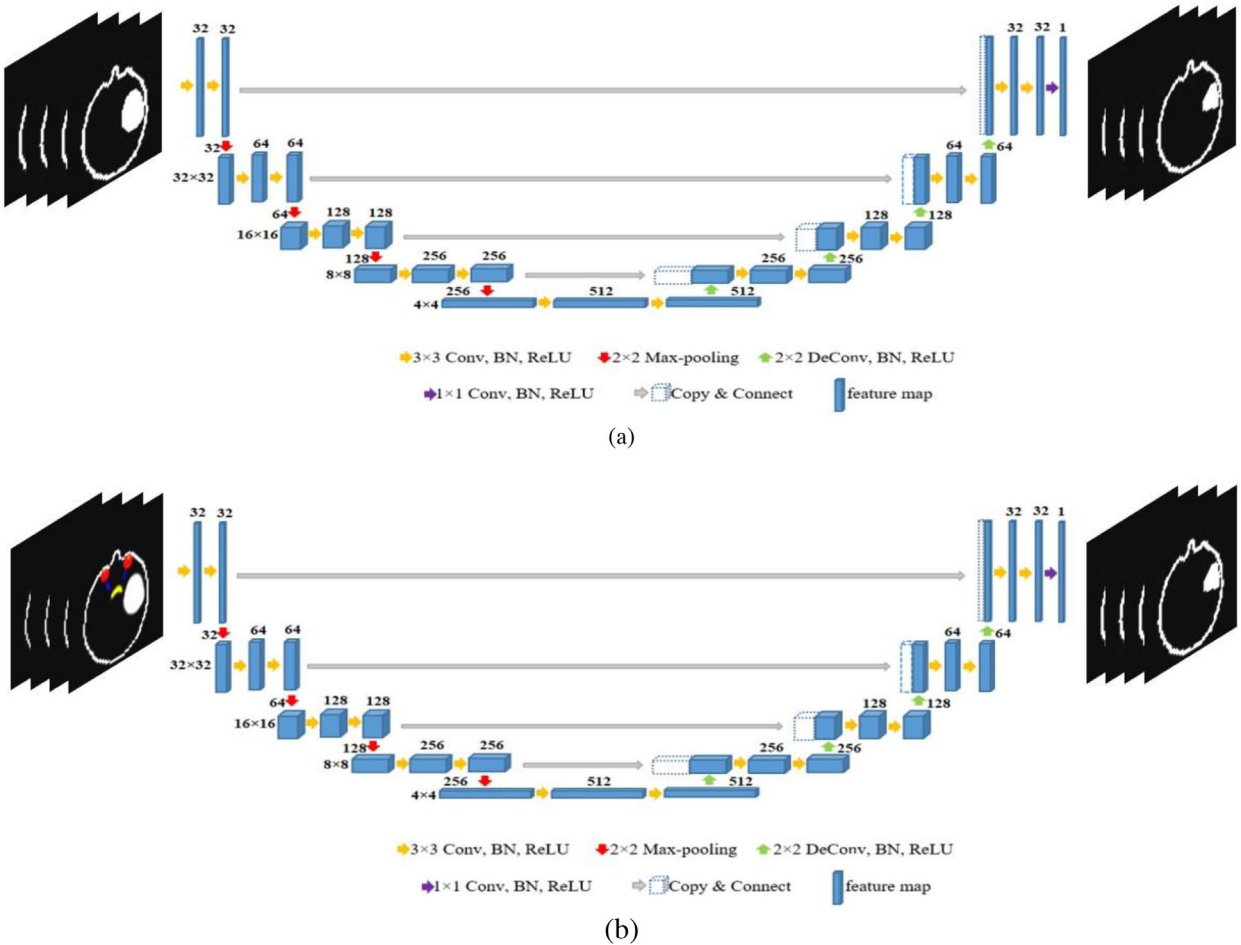
The schematic diagram for the training network of the proposed methods, (a) only PTV, (b) PTV‐OARs.

**FIGURE 2:**
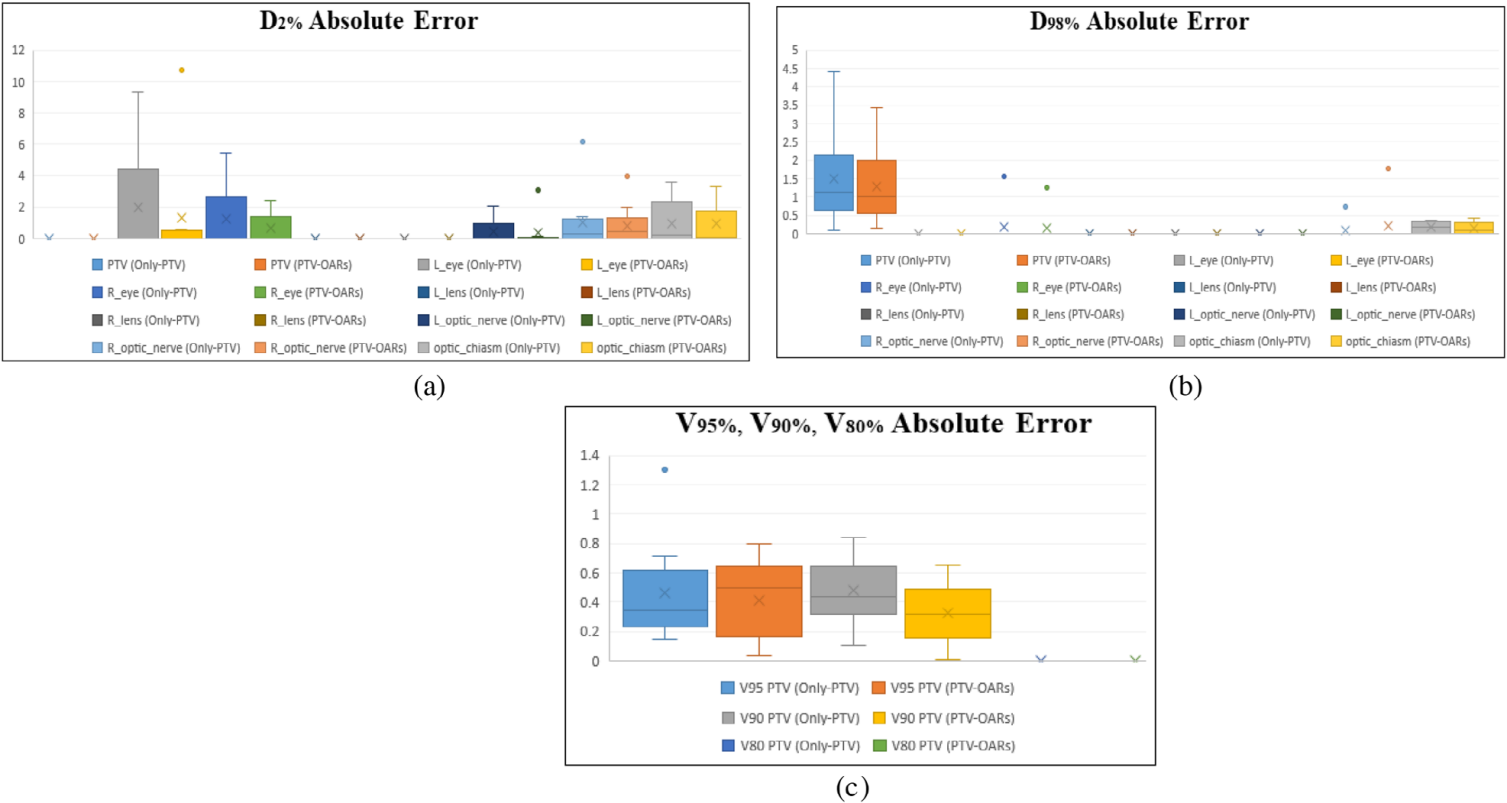
The box plot of the absolute error for various DVH parameters for the proposed methods versus the clinical dose of the test patients, (a) D_2%_ absolute error, (b) D_98%_ absolute error, (c) V_95%_, V_90%_, V_80%_ absolute error.

The IMRT images are usually stored in the DICOM format provided by TPS. First, using DICOM TO MATLAB software, various OAR contours and dose distributions were extracted and saved in BMP image format. Since a large number of images from different slices were regarded in the training set for higher resolution, memory occupation of the processing system increased during network training. Therefore, all images were stored and tested with a size of 64 × 64 × 3 for three‐channel images and 64 × 64 for two‐channel images. To prepare the training set employed in the first proposed method, from all slices relating to a patient, the slices containing PTV are selected and utilized as input of the U‐net network (Figure 1a). In the second method, in addition to the slices containing PTV, the slices which have the determined OARs are collected and then selected as input images (Figure 2a).

The corresponding dose slices of these images are also collected and regarded as targets and used in the network output for training. By placing the slices containing PTV, OARs, and respective Dose contours of each patient at the input and output of the models, 3D positional information is created for the network.

Since the dose prediction for different percentages of the prescribed dose was done separately in these methods, 10 training sets were prepared in each method. Additionally, for each training set, more than 5000 image slices were taken into account.

The training for the proposed methods ran for 100 epochs. We trained the network on a workstation with an Intel Core i7‐8700 CPU 3.7 GHz processor with 32 GB of RAM and a GeForce GTX 1080 Ti with 11 GB GPU memory.

## 3 RESULTS

In this research, the Only‐PTV method was first tested, therefore, only the PTV contour and also the Skin contour were introduced to the network as a distinguishing element helping to identify the location of the PTV in different brain regions and to reduce the computational load of the algorithm. The existence of only PTV and Skin contours made it possible to use black and white images, or in other words, two‐channel images. As you know, two‐channel images occupy less memory space than three‐channel RGB images. With the assurance of the results that the network is able to predict the dose distribution with only these elements, in order to increase the accuracy, four main organs with different colors in three channels form were applied to the input of the network in a separate test. As it was obtained from the results of the comparison of the two methods described in the following, there was no significant change in increasing the accuracy of the PTV‐OARs method, However, the only computational load of the network increased and showed itself in the time of network training.

The time spent for training the network in Only‐PTV method for 100 epochs by a computer with the specifications introduced above was about 25 min on average, while for the PTV‐OARs was almost 45 min.

In order to evaluate the accuracy of the network, in addition to visual criteria, four computational models have been applied to determine the similarity of the obtained images with the clinical dose images. These criteria are the accuracy determination by mean square error (MSE), dice metric, structural similarity index (SSIM), and dose‐volume histogram (DVH).

Dose distributions existed only in some brain CT image slices of a patient, and their shapes were different between various slices. Therefore, to evaluate the similarity between the predicted dose and the actual dose, the shape of the dose contour at the network output was also important, as well as, detecting the brain image slices and the position of the predicted dose in the scalp area.

Using mean square error (MSE), the overall difference between predicted dose images as X and clinical dose distribution images as Y are measured:

(1) $$MSE\;={\left(X-Y\right)}^{2}\;$$

Also, the dice metric^35^ which is one of the main metrics in semantic image segmentation is used to measure spatial overlap between predicted dose and clinical dose:

(2) $$Dice\;=\frac{2\left|X\cap Y\right|}{\left|X\right|+\left|Y\right|}\;$$

where ⋂ is the intersection between *X* and Y.

In another view, one of the criteria applied to determining the similarity of two images is the structural similarity index (SSIM).^36^ SSIM measures the similarity of two images based on the structure of natural images. The structure of natural images is such that voxels have a great dependence on their neighboring voxels, and this dependence contains important information about the structure of objects in the image. By calculating SSIM, the degree of structural similarity in the neighborhood of each individual voxel is calculated.

(3)

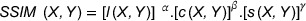

where *α* > 0, *β* > 0, *γ* > 0 denote the relative importance of each of the metrics and:

(4) $$l\;\left(X,Y\right)=\frac{2\mu_{x}\mu_{y}+C_{1}}{\mu_{x}^{2}+\mu_{y}^{2}+C_{1}}\;$$

(5) $$c\;\left(X,Y\right)=\frac{2\sigma_{x}\sigma_{y}+C_{2}}{\sigma_{x}^{2}+\sigma_{y}^{2}+C_{2}}\;$$

(6) $$s\;\left(X,Y\right)=\frac{\sigma_{xy}+C_{3}}{\sigma_{x}+\sigma_{y}+C_{3}}\;$$

where $\mu_{x}\;$and $\mu_{y}\;$are the local means, $\sigma_{x},\sigma_{y}\;$are the standard deviations and $\sigma_{xy}$ is the cross‐covariance for image patches *x, y*. If we assume, α = β = γ = 1 and C_3_ = C_2_/2, then Equ.3 is simplify to^32^:

(7) $$SSIM\;\left(X,Y\right)=\frac{\left(2\mu_{x}\mu_{y}+C_{1}\right)\left(2\sigma_{xy}+C_{2}\right)}{\left(\mu_{x}^{2}+\mu_{y}^{2}+C_{1}\right)\;\left(\sigma_{x}^{2}+\sigma_{y}^{2}+C_{2}\right)\;}\;.$$

The similarity of dose distribution images of the two proposed methods with respect to clinical doses based on MSE, dice metric, and SSIM for test patients are represented in Table 1.

**TABLE 1:** The average accuracy and similarity comparison of predicted dose distribution for test patients based on MSE, Dice, and SSIM.

Metrics	Test patients number	91	92	93	94	95	96	97	98	99	Total average
MSE	Only PTV	0.055	0.044	0.057	0.027	0.081	0.057	0.018	0.063	0.050	0.050
	PTV‐OARs	0.063	0.055	0.060	0.038	0.075	0.056	0.028	0.063	0.063	0.056
Dice	Only PTV	0.827	0.882	0.835	0.923	0.752	0.784	0.950	0.837	0.870	0.851
	PTV‐OARs	0.801	0.879	0.835	0.892	0.780	0.791	0.948	0.827	0.825	0.842
SSIM	Only PTV	0.84	0.82	0.82	0.88	0.78	0.81	0.9	0.8	0.83	0.83
	PTV‐OARs	0.85	0.81	0.78	0.84	0.78	0.82	0.89	0.78	0.81	0.82

These results show a degree of high accuracy between the dose predicted by the proposed methods for the test patients and the clinical does distributions obtained from TPS.

To check the performance of the proposed method accurately in line with clinical criterion, the DVH parameters of all the test patients were calculated and their absolute error in comparison with clinical dose distribution with significant differences (*p*‐value <0.05) are measured in Table 2. In this table, the absolute errors of V_95%_, V_90%_, and V_80%_ for PTV are calculated. Additionally, the absolute errors of minimum and maximum dose received by PTV and all OARs (D_2%_ and D_98%_) are obtained. The box plot of absolute error for various DVH parameters of the proposed methods versus the clinical doses for the test patients including V_80%_, V_90%,_ V_95%_, D_2%_, and D_98%_ for PTV and OARs are calculated and illustrated in Figure 2.

**TABLE 2:** The absolute error for various DVH parameters of the proposed methods compared to the clinical dose for the test patients.

PTV and OARS	DVH parameter	Only‐PTV (Mean + Variance)	OARs‐PTV (Mean+Variance)	*p*‐Value
PTV	V_80%_	0	0	0
PTV	V_90%_	0.47 + 0.05	0.32 + 0.04	0.16
PTV	V_95%_	0.45 + 0.13	0.40 + 0.07	0.74
PTV	D_98%_	1.5 + 1.72	1.29 + 1.06	0.71
L_EYE	D_98%_	0	0	0
	D_2%_	2.0 + 15.6	1.30 + 12.57	0.69
R_EYE	D_98%_	0.17 + 0.26	0.13 + 0.17	0.87
	D_2%_	1.2 + 3.87	0.61 + 0.83	0.42
L_LENS	D_98%_	0	0	0
	D_2%_	0	0	0
R_LENS	D_98%_	0	0	0
	D_2%_	0	0	0
L_OPTIC_NERVE	D_98%_	0	0	0
	D_2%_	0.43 + 0.76	0.35 + 1.04	0.85
R_OPTIC_NERVE	D_98%_	0.08 + 0.05	0.19 + 0.34	0.59
	D_2%_	1.05 + 3.97	0.82 + 1.75	0.78
OPTIC_CHIASM	D_98%_	0.16 + 0.02	0.14 + 0.02	0.8
	D_2%_	0.97 + 1.98	0.93 + 1.43	0.93

This table also shows the high accuracy prediction of the proposed methods compared to the clinical doses.

**FIGURE 3:**
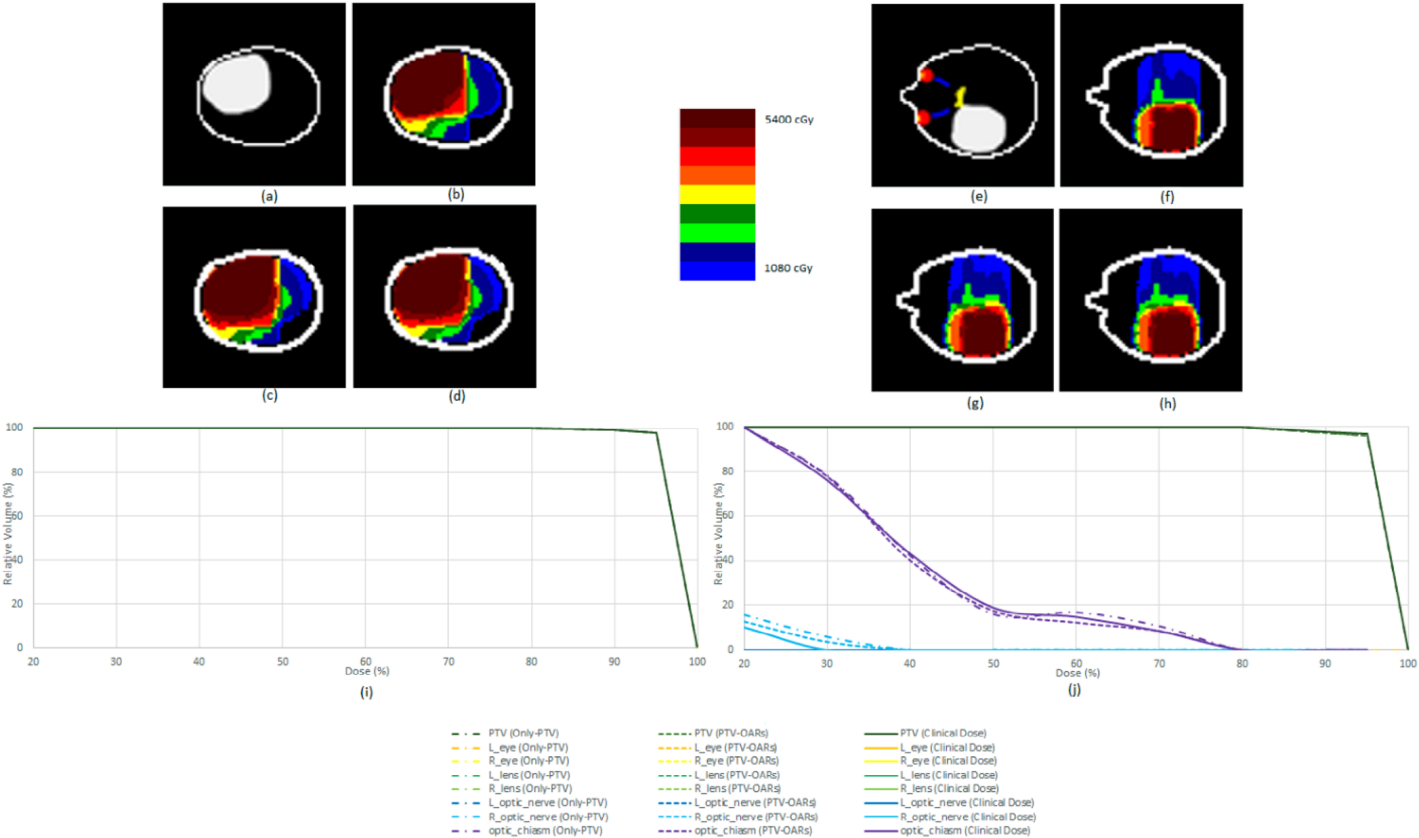
Visual evaluation of the predicted dose distribution for 94th patient in right, (a) PTV (network input image), (b) clinical dose distribution, (c) predicted dose distribution by only‐PTV method, (d) predicted dose distribution by PTV‐OARs method and for 98th patient in left, (e) PTV+OARs (network input image), (f) clinical dose distribution, (g) predicted dose distribution by only‐PTV method, (h) predicted dose distribution by PTV‐OARs method, (i) DVH plot 94th patient, (j) DVH plot 98th patient.

Since the accuracy for various test patients are different and noting the fact that the tumors may occur in various positions of the brain and also their distances from the OARs are different, TPS may create different dose distributions due to the OARs limitations. According to the distribution of OARs in different parts of the brain, if the training images are not homogeneous in number, type, and location of the tumors during network training, the network may be better trained and have better results for some tumors than others. To discuss this issue further, the visual comparisons of the images of the two patients who had tumors in different parts of the brain are depicted in Figure 3a,e. In addition, the DVH diagrams which described the effects of doses on PTV and OARs are shown in Figure 3i,j. In both analyses, it is clear that the high accuracy of our proposed methods is comparable to the clinical dose distributions designed by TPS.

Also in Table 3, the DVH parameters of the proposed methods with the clinical doses of these two patients, V_80%_, V_90%_, V_100%_, and D_98%_ for PTV and D_2%_ and D_98%_ for OARs are represented. This table shows the high prediction accuracy and compatibility of the proposed method with the clinical doses. With respect to the recent results, we can conclude that some of the OARs were affected by radiation and absorbed the dose in one patient but in another case, none of the OARs were affected by the treatment dose.

**TABLE 3:** The comparison of proposed methods with clinical dose based on the volume of PTV covered by different percentage doses and volume of OARs covered by maximum and minimum percentage doses for 94th and 98th patients.

		94th patient			98th patient		
PTV and OARS	DVH parameter	Only‐PTV	OARs‐PTV	Clinical Dose	Only‐PTV	OARs‐PTV	Clinical dose
PTV	V_80%_	100	100	100	100	100	100
PTV	V_90%_	99.24	99.35	99.34	97.43	97.37	97.82
PTV	V_95%_	98.03	98.14	98.17	96.08	96.1	96.6
PTV	D_98%_	93.22	93.02	92.27	89.11	89.02	90.24
L_EYE	D_98%_	0	0	0	0	0	0
	D_2%_	0	0	0	0	0	0
R_EYE	D_98%_	0	0	0	0	0	0
	D_2%_	0	0	0	0	0	0
L_LENS	D_98%_	0	0	0	0	0	0
	D_2%_	0	0	0	0	0	0
R_LENS	D_98%_	0	0	0	0	0	0
	D_2%_	0	0	0	0	0	0
L_OPTIC_NERVE	D_98%_	0	0	0	0	0	0
	D_2%_	0	0	0	0	0	0
R_OPTIC_NERVE	D_98%_	0	0	0	0	0	0
	D_2%_	0	0	0	37.67	35.42	31.48
OPTIC_CHIASM	D_98%_	0	0	0	21.5	21.48	21.31
	D_2%_	0	0	0	80.57	75.35	77

## 4 DISCUSSION

Our analyses showed that applying the deep learning method had a high ability to predict the dose distribution of the IMRT TPS for brain tumors (Table 1). According to the DVH parameters represented in Table 2 we can see the average difference between clinical results with the Only‐PTV method for PTV is about 0.45% with 0.13% variance and for PTV‐OARs in V_95%_ is about 0.4% with 0.07% variance. These average differences for PTV in V_90%_ are about 0.47% with 0.05% variance for the Only‐PTV method and about 0.32% with 0.04% variance for the PTV‐OARs method. The minimum and maximum average difference in D_2%_ for OARs between the two proposed methods and clinical doses are (0.43%–2.0%) for the Only‐PTV method and (0.35%–1.3%) for the PTV‐OARs method. The same comparison in D_98%_ for OARs shows (0.08%–0.17%) deviation for the Only‐PTV method and (0.13%–0.17%) for the PTV‐OARs method. According to these results, there is a significant accordance between the obtained results of our proposed methods and the clinical doses. To our knowledge, no research has been conducted to predict the dose distribution for brain tumors using IMRT images by deep learning. Therefore, it is difficult to compare the current results directly with the works done on other organs and datasets,^23^, ^24^, ^25^, ^26^, ^27^, ^28^, ^29^, ^30^ but our overall results on the use of deep learning in dose prediction are comparable to the results of the above research. In ref.,^23^ to predict the dose distribution for prostate cancer by IMRT applying a U‐net model, the range of errors for the PTV and OAR parameters were reported ±8.00 and ±18.0%, respectively. Also, in a similar research in the same field,^25^ an accuracy of 9.95 × 10^−4^ and 0.994 was obtained for image data based on the mean absolute error (MAE) and SSIM criteria.

The following results have been obtained from the researches^32^, ^33^, ^34^ conducted on the OpenKBP challenge dataset to treat head and neck cancer. Also in these researches, modified U‐net models have been used to increase accuracy. By using a 3D cascade U‐Net in ref.^32^ the MAE difference between the predicted and real dose distributions 2.31 was gained. The dose prediction difference between 3D cascade U‐Net and the U‐net model is about 0.28 to 1.96 Gy for PTV and OARs; however, using a 3D dense dilated U‐net architecture^33^ makes the MAE to be equivalent to 2.56 Gy. In the comparison between the attention U‐net and U‐net model in ref.^34^ to predicted IMRT dose for head and neck cancer, the accuracy of MAE 2.43 Gy versus 2.81 was obtained for the body contour. However, the difference in accuracy for PTV and OARs tested in this research between (−0.61–1.47) Gy based on MAE with respect to clinical dose was expressed. In other words, in some OARs, and PTV, attention U‐net had better results. In another analysis that we discussed in this article, we sought to use two different patients who had tumors in different areas (one in the temporal lobe (middle and anterior of the head) and one in the posterior fossa (back of the head)) to assess multiple targets. In another view, the proposed methods were also evaluated by images containing only PTV, as well as cases that contain PTV and OARs.

First, the presence of the tumors in various parts and their distances from the OARs, has caused TPS to design a dose distribution that has the least effect on the OARs (Figure 3). But in Figure 3a, where the tumor is far from OARs respect to, Figure 3e the treatment planning system designed the dose distribution more freely. These effects are clearly visible in DVH curves (Figure 3i,j) and also in Table 3. The point that can be mentioned in this section is that in some areas of the brain, due to the lack of information, the network has not trained well. Therefore, these can lead to different results in terms of accuracy for various test patients. However, in our proposed method, according to the employed images of the training set, the accuracies are mostly acceptable, but in the obtained results for the test patients (Tables 1 and 2), the difference in accuracies is visible. Thus, in order to achieve higher accuracy, separating the brain areas based on the distance of PTVs to OARs and collecting the necessary large number of images for each category can lead to better results. Hence to achieve higher accuracy for future works, it is recommended to predict the dose separately for different parts of the brain using deep learning.

The second point that can be discussed here is the slight difference between the results of the two proposed methods namely only PTV and PTV‐OARs. By experience, we have found that prescribed dose distributions (designed by TPS) are usually in the slices in which PTV is defined for them. In other words, when a treatment plan is designed for a patient, the dose distribution created in each slice is based on the OARs limitations. It means the knowledge related to the existence of these limitations is present in the dose distribution by itself (Figure 1a,b) and the dose distribution here is the output of the network. So if the PTV alone is introduced at the network's input, and the slice type is also specified, redefining the OARs does not create new knowledge for the network, because, in our proposed methods, dose distribution prediction was not based on the OARs restrictions. In other words, in our proposed methods, we seek to acquire knowledge through deep learning which has been employed in the treatment of previous patients and exists in their therapeutic images. By gaining knowledge from the treatment of previous patients, we want to increase the speed of treatment planning for new patients so that the quality of treatment does not change substantially. The obtained results practically prove the mentioned hypothesis.

Therefore, the PTV and skin contour are not only able to predict the doses of the tested patients, but this method has an accuracy equivalent to the second method, while the computational load of the network has been reduced by half based on the training time of the network. This is in the case that in all references studied in the introduction section,^23^, ^24^, ^25^, ^26^, ^27^, ^28^, ^29^, ^30^, ^31^, ^32^, ^33^, ^34^ the contours of the OARs are considered in the input of networks. This research also states that by using the only‐PTV method, the introduction time of OARs is reduced and the computational load of the training network is decreased. Also, the importance of the skin contour is more clearly defined, because its various shapes in different slices can indicate the existence of OARs such as the eyes, lenses, optic nerves, etc., for the network indirectly.

The main emphasis of the authors in this article, besides the feasibility of predicting the dose distribution of brain tumor treatment with IMRT using deep learning, has been to reduce the information that has redundant role in the network, which has reduced the speed of the network. It seems useful to remember this point that in deep learning, feature extraction is achieved by applying a large set of images. In this research, due to the presence of many images and memory limitations, small size 64 × 64 images were used instead of the original images. Therefore, in order to increase the accuracy of the network, it is very important to reduce the additional information that allows feeding the network with more images. Of course, transfer learning can also be one of the solutions.

However, in the PTV‐OARs method, in addition to the PTV, all the slices that had the OARs, also have been introduced to the network while PTV may not be present in those images. Therefore, small differences in the input images can cause small differences in the obtained accuracies of the results.

Therefore, in our methods, the network learns to provide the same dose distribution for the treatment of new patients based on various PTVs in different brain image slices existing in the training set. Hence the only‐PTV method, which removes the process of introducing OARs, can significantly increase the speed of the dose distribution designing process with respect to the PTV‐OARs method Applying the new U‐net model to improve the accuracy and using automated OARs segmentation based on deep learning to increase the speed of treatment planning is suggested for future work for the brain tumors.

## 5 CONCLUSION

The goal of training‐based dose prediction algorithms like deep learning is to gain applied knowledge in the treatment planning of previous patients and employ it in future planning to increase the speed of algorithms. In this article, the accuracy of dose prediction designed by IMRT for brain tumors using deep learning and its effect on treatment planning time was investigated. In the next step, the performances of dose prediction accuracy in cases where PTV is defined alone or PTV is introduced to the network as well as OARs were studied. The almost identical accuracy of the two approaches showed that the presence of OARs in addition to PTV does not provide new knowledge to the network. Accordingly, the Only‐PTV method by eliminating the process of defining OARs reduces the overall time of the treatment planning process.

## AUTHOR CONTRIBUTIONS

Maziar Irannejad: Designed the model and the computational framework and analyzed the data. Iraj Abedi: Physicist, proposing the initial idea, providing the data set to be tested and also investigating the results of the proposed method with clinical results. Vida Darbaghi Lonbani: Pre‐processed the data set and performed the computations. Maryam Hassanvand: developed the theory, helped supervise the project, and wrote the manuscript. All authors discussed the results and contributed to the final manuscript.

## CONFLICT OF INTEREST STATEMENT

The authors declare that they have no known competing financial interests or personal relationships that could have appeared to influence the work reported in this paper.

## ETHICS STATEMENT

This research was approved by the Research Ethics Committee of the Vice‐Chancellor in Research Affairs‐Medical University of Isfahan, on 22.02.2022. Approval ID: IR.MULRESEARCH.REC.1400.471. https://ethics.research.ac.ir/ProposalCertificateEn.php?id=248597&Print=true&NoPrintHeader=true&NoPrintFooter=true&NoPrintPageBorder=true&LetterPrint=true

## Data Availability

Research data are stored in an institutional repository and will be shared upon request with the corresponding author.
